# Supplementary material for: Ligand-Mediated Biofilm Formation via Enhanced Physical Interaction between a Diguanylate Cyclase and Its Receptor
Source: mBio. 2018 Jul 10;9(4):e01254-18. doi: 10.1128/mBio.01254-18 (PMC6050961; doi:10.1128/mBio.01254-18)
Supplement: FIG S1 [file mbo004183974sf1.pdf]

```
GcbC            REHANAQESATRGATTIAQL---IDADVLRTVELYDLTLQGLIAAAQRDDLKDVSPQIRH
Z-16            KGYDSHKIALAQSETEMRNLSHSLAEHATHTFQGADVVLDDIVSFMKWRPHPSPVFNERL
                . : . : : :.. * : :*   :   . .*.:  *:.*:.:::  :.    .   : *

GcbC            LALFDRSTTARFKGDILLLDKHGEVIADSSRVDPLPG-NFADRDYFLAHAFNRDTGMFIS
Z-16            RALADNLPQL---SDVAILDADGQLTYAS--VKPVPALDNSDRSYFRYHRANDDHTLLIT
                 ** *. .     .*: :**  *::   *  *.*:*. : :**.**  *  * *  ::*:

GcbC            RPFKPRCDCDDADQWRISFSRRISSNTGEFLGVAVASMKLDYFDQLFNSLDIGIDSTLNI
Z-16            GPIQSRT----SGVWVFVVSRRLETTDGKFFGVVVATIESEYFSTFYKTFDLGPGGSISL
                 *::.*     :. * : .***:.:. *:*:**.**::: :**. :::::*:* ..::.:

GcbC            INNDGILLAQKPYLQSDSIGKSFAARPNVVRILRDSSGNGSFNSISSMDHQQRLYTYSRV
Z-16            LHSDGRLLIQWPSLQT---GRDMANMVLFQKALPRSP-DGYYLTVSPFDGLTKYLAYRRV
                ::.** ** *.* **:   *..:*    . . *  *. :* : ::*.:*   .  :* **

GcbC            GNLPLTVIVALSSEEVFGAWR------
Z-16            SRYPLVVTVARTEDSVLSGWREAVRSD
                .. **.* ** :.:.*:..**
```

Fig. S1. CLUSTAL alignment of the amino acid sequence of the CACHE domain of GcbC and rpHK1S-Z16 (PDB ID: 3LIF) of *R. palustris*. The CACHE domain of GcbC and rpHK1S-Z16 showed 31% identity at the amino acid level (E-score = $1e^{-27}$). The red amino acids indicate the conserved residues in the proposed citrate-binding pocket. A subset of these residues are mutated (see Figures 2 and 3 in the main text, and Table S1).
