# Supplementary material for: Ligand-Mediated Biofilm Formation via Enhanced Physical Interaction between a Diguanylate Cyclase and Its Receptor
Source: mBio. 2018 Jul 10;9(4):e01254-18. doi: 10.1128/mBio.01254-18 (PMC6050961; doi:10.1128/mBio.01254-18)
Supplement: TABLE S1 [file mbo004183974st1.pdf]

Table S1. CACHE Domain Mutations.

Mutation	Amino Acid Number	Stable[a]
R to A	139	Yes
R to E	139	Yes
Y to F	141	No[b]
R to A	162	Yes
ΔCACHE	113 - 297	No
R to A	67	No
R to A	79	No
R to K	139	No
R to S	139	No
D to S	140	No
D to E	140	No
Y to D	141	No
H to A	145	No
F to L	154	No
R to E	162	No
D to G	169	No
R to A	172	No
R to E	172	No
S to A	176	No
G to A	184	No
G to A	188	No
V to A	189	No
R to A	247	No
S to G	253	No
V to M	292	No
S to N	304	No
DY to AA	140 – 141	No
DY to AA	197 – 198	No
DYF to SFF	140 – 142	No
DYF to LDS	197 - 199	No
RDYF to LLDS	139 - 142	No

[a]As assessed by Western blot.

[b]While we could detect the GcbC protein for the Y141F mutant it was much less stable than the WT.
