# Supplementary material for: Ligand-Mediated Biofilm Formation via Enhanced Physical Interaction between a Diguanylate Cyclase and Its Receptor
Source: mBio. 2018 Jul 10;9(4):e01254-18. doi: 10.1128/mBio.01254-18 (PMC6050961; doi:10.1128/mBio.01254-18)
Supplement: FIG S2 [file mbo004183974sf2.pdf]

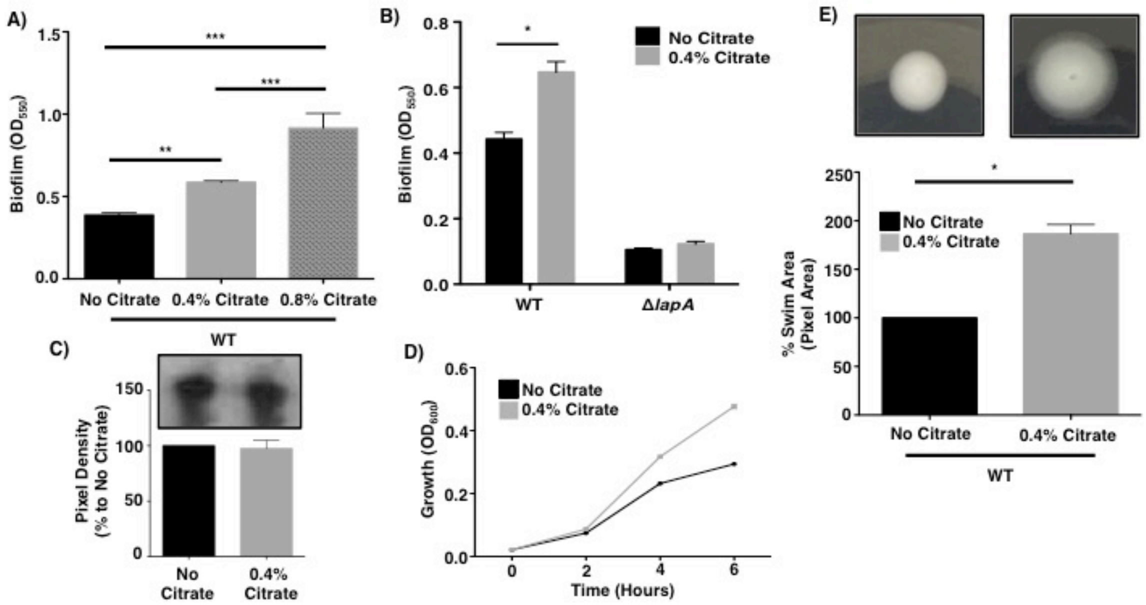

Fig. S2. Effect of a *lapA* mutation on citrate-mediated biofilm formation. (A) Quantitative analysis of dose-dependent biofilm formation by WT *P. fluorescens* by addition of increasing concentrations of citrate. Citrate concentrations of 0%, 0.4%, and 0.8% were tested. (B) Quantitative analysis of biofilm formation by the WT and *lapA* mutant of *P. fluorescens* in the presence and absence of citrate. (C) Quantification of LapA levels in whole cell lysate in the presence and absence of 0.4% citrate by WT *P. fluorescens* Pf0-1. Representative blots are shown. Data shown is the average of three replicates (+ SD). (D) Growth of WT *P. fluorescens* in the presence and absence of citrate for 6 hours at 30°C. (E) Motility by WT *P. fluorescens* in the presence and absence of citrate at 30°C for 30 hours using a soft agar (0.3%) motility assay. Representative images are shown. In all experiments in this figure, assays were performed in triplicate (+ SD), and horizontal black bar indicates a P value of <0.05 (*), <0.01 (**), or <0.001 (***) by a student's t-test comparing each strain without citrate versus with added citrate, or in panel A, comparing the two different citrate concentrations tested.
