# Supplementary material for: Ligand-Mediated Biofilm Formation via Enhanced Physical Interaction between a Diguanylate Cyclase and Its Receptor
Source: mBio. 2018 Jul 10;9(4):e01254-18. doi: 10.1128/mBio.01254-18 (PMC6050961; doi:10.1128/mBio.01254-18)
Supplement: FIG S3 [file mbo004183974sf3.pdf]

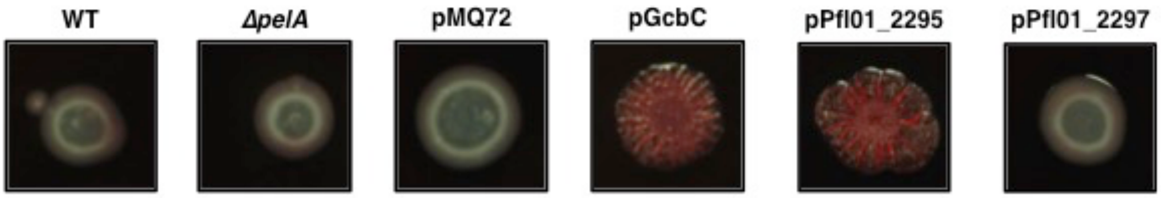

Fig. S3. Assessment of DGC activity via congo red binding. Qualitative analysis of GcbC, Pfl01_2295, and Pfl01_2297 to test for functional c-di-GMP production as described in the main text. These plasmids were transformed into *P. aeruginosa* PA14, and the strains grown on congo red plates supplemented with 0.1% arabinose at 37°C to induce expression of the plasmid-borne DGCs for 24 hours. The binding of red pigment indicates Pel exopolysaccharide production, which serves as an indirect means to assess the amount of c-di-GMP produced by the indicated strain. GcbC and Pfl01_2295 show a higher degree of congo red binding as compared to the empty pMQ72 control and the *pelA* deletion mutant strain. Pfl01_2297 showed only a very modest enhancement of congo red binding compared to the empty pMQ72 control and *pelA* deletion mutant strain.
