# Supplementary material for: Ligand-Mediated Biofilm Formation via Enhanced Physical Interaction between a Diguanylate Cyclase and Its Receptor
Source: mBio. 2018 Jul 10;9(4):e01254-18. doi: 10.1128/mBio.01254-18 (PMC6050961; doi:10.1128/mBio.01254-18)
Supplement: FIG S4 [file mbo004183974sf4.pdf]

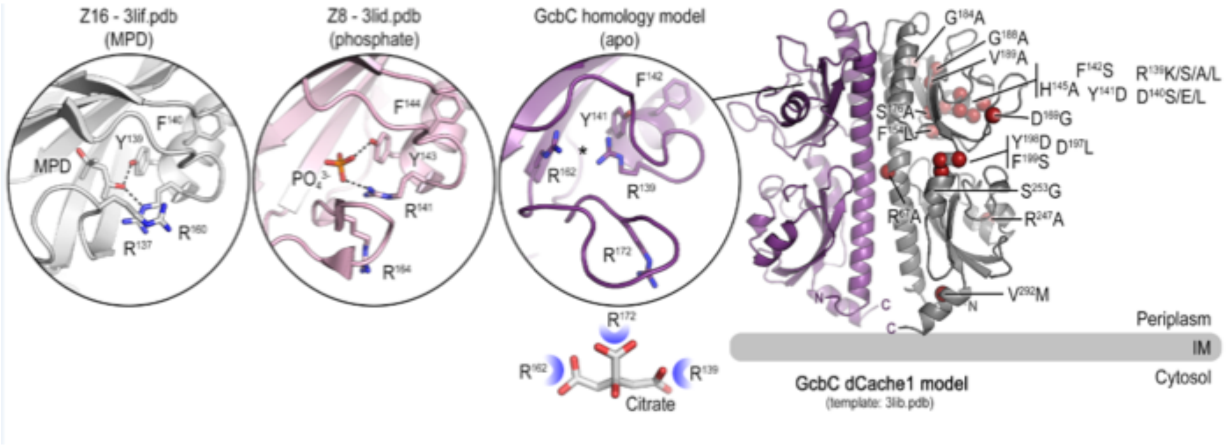

Fig. S4. M Modeling the CACHE domain of GcbC on rpHK1S-Z16 (PDB ID: 3LIF) and vpHK1S-Z8 (PDB ID: 3LID). Shown is the model of the ligand-binding site of rpHK1S-Z16 (PDB ID: 3LIF) and vpHK1S-Z8 (PDB ID: 3LID), as well as the homology model of the ligand-binding site of the apo-GcbC. On the far right is shown the overall model of the GcbC periplasmic domain based on the structure of rpHK1S-Z16 (PDB ID: 3LIF).
