# Supplementary material for: Ligand-Mediated Biofilm Formation via Enhanced Physical Interaction between a Diguanylate Cyclase and Its Receptor
Source: mBio. 2018 Jul 10;9(4):e01254-18. doi: 10.1128/mBio.01254-18 (PMC6050961; doi:10.1128/mBio.01254-18)
Supplement: FIG S5 [file mbo004183974sf5.pdf]

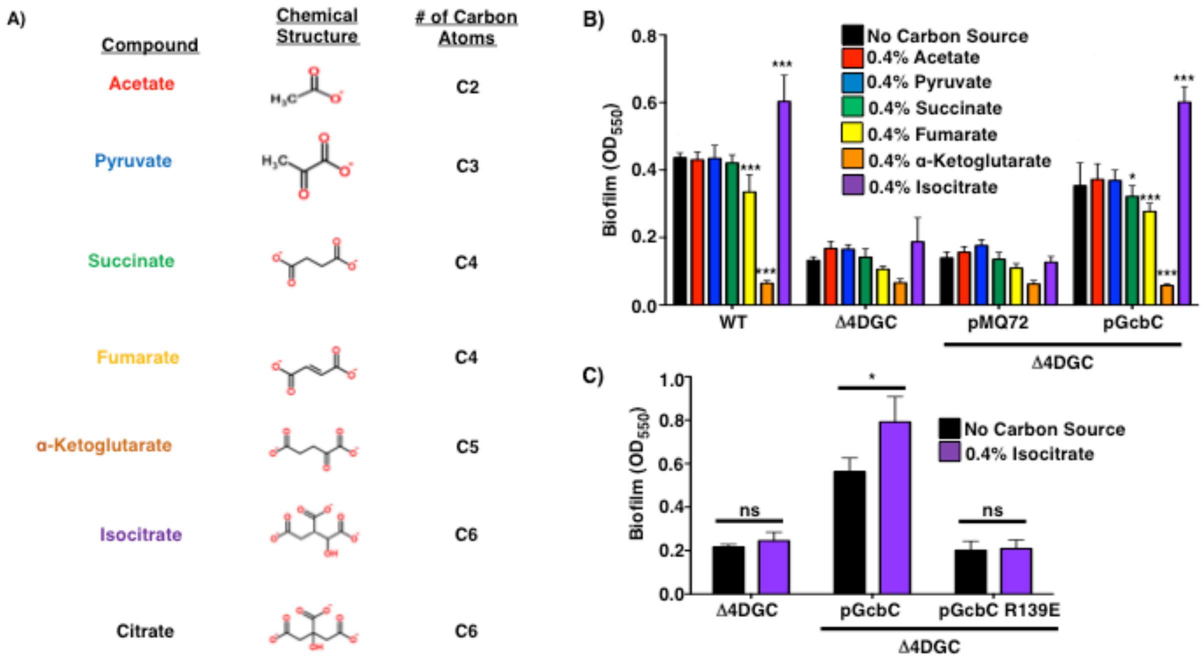

Fig. S5. Identification of ligands sensed by GcbC's CACHE domain. (A) Shown is a list of organic acids that are color coordinated with the graph in panel B. The chemical structure of acetate, pyruvate, succinate, fumarate, α-ketoglutarate, isocitrate, and citrate are shown. The number of carbon atoms of each compound is listed on the right. (B) Quantitative analysis of biofilm formation by WT *P. fluorescens* and the Δ4DGC mutant strain in the presence of 0.4% acetate, pyruvate, succinate, fumarate, α-ketoglutarate, and isocitrate. The empty vector control and a plasmid carrying GcbC were introduced into the Δ4DGC mutant strain. Biofilm assay data are from six biological replicates (± SD). Linear models implemented in R (36) were used to identify organic acid supplemented media whose properties significantly differed from the base medium (K10T-1 minimal medium, see Materials and Methods) for both biofilm assay and B2H assay experiments. The reduced biofilm levels for medium supplemented with α-ketoglutarate

is due to the poor growth of the strains in the presence of this compound (not shown). P values of <0.05 were considered significant. P<0.05 (*); P<0.001 (***). (C) Biofilm formation by the indicated strains ± isocitrate. The Δ4DGC mutant strain is used with WT GcbC and GcbC-R139E variant introduced on plasmids. For panel C, experiments were performed in triplicate (± SD), and horizontal black bars indicate a P value of <0.05(*) with a student's t-test comparing the presence and absence of isocitrate amongst each strain.
