# Supplementary material for: Ligand-Mediated Biofilm Formation via Enhanced Physical Interaction between a Diguanylate Cyclase and Its Receptor
Source: mBio. 2018 Jul 10;9(4):e01254-18. doi: 10.1128/mBio.01254-18 (PMC6050961; doi:10.1128/mBio.01254-18)
Supplement: FIG S6 [file mbo004183974sf6.pdf]

```
GcbC            -------REHANAQESATRGATTIAQLIDADVLRTVELYDLTLQGLIAAAQRDDLKDVS-
Pfl01_2295      ----SRERALEEVDVHGLNLTQALVTYSEGIVRQS----SALLLGLVERLETEGSGPVQI
Pfl01_2297      ------RSVFASARDSVTNLARATAQHAEDTIRQV----DVLTAGLAERVEGDGLQNLDV
Pfl01_3800      QIEQSRRQDLANAQVSSANLTRAMAQQAEDTFLAA----DLVMTSLVDWVQEDGYGAAQK
                           ..     . : : .   :  .       .    .*    : :.    .

GcbC            PQIRHLALFDRSTTARFKGDILLLDKHGEVIADSSRVDPLPGNFADRDYFLAHAFNRDTG
Pfl01_2295      QRISSLIDRQEPLMPQLSG-ITIYDSQGRWLMSSNRPIPAGANSSDRAYFIHHRDDPSPE
Pfl01_2297      ARIHKLLVQQSKIMPQLHG-LFIYGPDGHWIVTDKEVTPETANNADRDYFQYHRTHEDRR
Pfl01_3800      PRLQRTFARRVQQLEQLHG-MFLFDREGQWVITSFPDLPRGNGVADREYFKFHQQNVSGV
                 .:            .: * : : .  *  :  .    *   . :** **  *    .

GcbC            MFISRPFKPRCDCDDADQWRISFSRRISSNTGEFLGVAVASMKLDYFDQLFNSLDIGIDS
Pfl01_2295      TFIGPPIQSRTNQE----WVITVSRRFNDARGEFAGVVAVTLGIENFLRLFGKIDVGQEG
Pfl01_2297      VRIGQVVESRSTHD----LIIPISRRLNNPDGSFAGVLLGTVKVSYFVDYYGDFRIDDKG
Pfl01_3800      AHIGPAIRSRENGE----WIIPISKRVNDRAGNFQGVLLAGIKMSYFDKFFKSFSLDDNG
                  *.  . .*   :      *..*.*...  *.* **    : :. *   : .: :. ..

GcbC            TLNIINNDGILLAQKPYLQSDSIGKSFAARPNVVRILRDSSGNGSFNSISSMDHQQRLYT
Pfl01_2295      AIGLSYTDGTLLVRYPFREQD-MGRNFSKSPIYAKYLVDHS-VGTASFTSSLDGVERLYA
Pfl01_2297      ALVLAMRDGTILVRRPFITSV-VGKSLANSEIFKTYLPNAN-QGIVQIRAVVDDTERLYG
Pfl01_3800      TMFLGMTDGTLLARRPFDESL-IGTSLAKGEIYQKLLPNAS-AGTAMIDSVVDGVTRLYG
                :: :   ** :*.. *:  .  :* .::        * : .  *     : :*   ***

GcbC            YSRVGNLPLTVIVALSSEEVFGAWR---
Pfl01_2295      FRKSDRLPLITTVALGKREALAAWRTEA
Pfl01_2297      YRALTTYPLVVEAGLSRDSIVAPWRQ—
Pfl01_3800      YRQLESYPLVVAASSSRDTILQGWYDR-
                :      ** . .. .    .  *
```

Fig. S6. CLUSTAL alignment of the amino acid sequence of the CACHE domains of GcbC, Pfl01_2295, and Pfl01_2297. The red amino acids indicate the conserved residues of the RXYF motif. The residues highlighted in blue are proposed to make up the putative citrate-binding pocket of the CACHE domain of GcbC. The RXYF motif and residues R139 and R162 of GcbC are conserved in the CACHE domains of Pfl01_2295, Pfl01_2297, and Pfl01_3800.
