# Supplementary material for: Ligand-Mediated Biofilm Formation via Enhanced Physical Interaction between a Diguanylate Cyclase and Its Receptor
Source: mBio. 2018 Jul 10;9(4):e01254-18. doi: 10.1128/mBio.01254-18 (PMC6050961; doi:10.1128/mBio.01254-18)
Supplement: FIG S7 [file mbo004183974sf7.pdf]

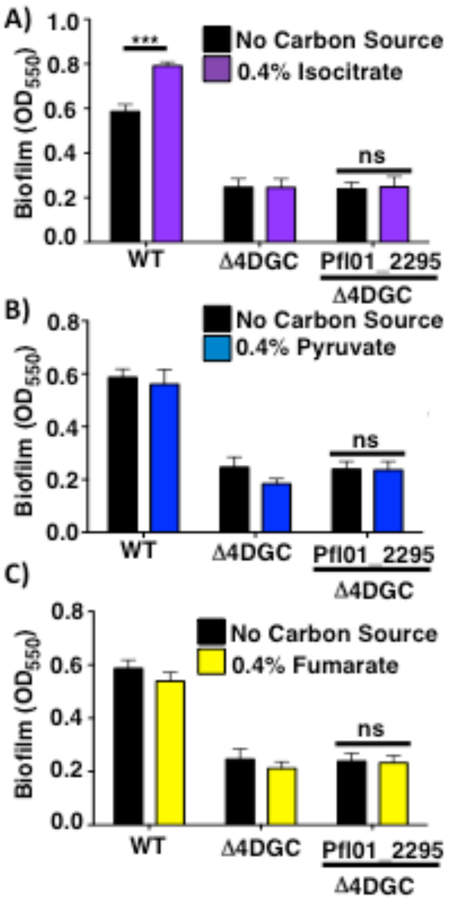

Fig. S7. Identification of potential ligands sensed by Pfl01_2295. Shown are the results of biofilm assays with the indicated strains. For each panel, the presence and absence of each organic acid was compared for each biofilm assay. Panel A = +/- isocitrate; B = +/- pyruvate; C = +/- fumarate. Biofilm assays are representative of six biological replicates P<0.001 (***).
