# Supplementary material for: Ligand-Mediated Biofilm Formation via Enhanced Physical Interaction between a Diguanylate Cyclase and Its Receptor
Source: mBio. 2018 Jul 10;9(4):e01254-18. doi: 10.1128/mBio.01254-18 (PMC6050961; doi:10.1128/mBio.01254-18)
Supplement: TABLE S2 [file mbo004183974st2.pdf]

Table S2. Strains, Plasmids, and Primers Used in this Study.

Strain	Genotype or Description	Reference
Saccharomyces cerevisiae		
InvSc1	MATa/ MATα leu2/ leu2 trp1-289/trp1-289 ura3-52/ura3-	Invitrogen
Escherichia coli		
S17-1-λ-pir	*thi pro hsdR- hsdM* + Δ*recA* RP4-2::TcMu-Km::Tn7	1
BTH101	F-, *cya-99*, *araD139, galE15, galK16, rpsL1 (Str r)*, *hsdR2, mcrA1, mcrB1.*	Euromedex
Pseudomonas aeruginosa		
SMC 232	Wild type PA14	2
SMC 2893	Δ*pelA*	3
SMC 7218	pMQ72	This Study
SMC 7219	pMQ72 GcbC	This Study
SMC 6150	pMQ72 Pfl01_2295	This Study
SMC 6149	pMQ72 Pfl01_2297	This Study
Pseudomonas fluorescens		
SMC 4798	Wild Type, HA-tagged LapA variant	4
SMC 4754	SMC 4798 with unmarked deletion of Δ*gcbC*	5
SMC 4945	Wild Type + pMQ72 GcbC-HA	This Study
SMC 4946	Wild Type + pMQ72 GcbC (GGAAF)-HA	This Study
SMC 7288	Δ*lapA*	This Study
SMC 6202	SMC 4798 with unmarked deletion of Δ*gcbA* Δ*gcbB* Δ*gcbC* Δ*wspR* (Δ4DGC)	5
SMC 7211	Δ4DGC + pMQ72 GcbC-HA	This Study
SMC 7213	Δ4DGC + pMQ72 GcbC (GGAAF)-HA	This Study
SMC 7699	Δ4DGC + pMQ72	This Study
SMC 7701	Δ4DGC + pMQ72 GcbC R139A-HA	This Study
SMC 7702	Δ4DGC + pMQ72 GcbC R139E-HA	This Study
SMC 7703	Δ4DGC + pMQ72 GcbC R162A-HA	This Study
SMC 6154	Δ4DGC + pMQ72 Pfl01_2295	6
SMC 6153	Δ4DGC + pMQ72 Pfl01_2297	This Study
**Plasmids**		
pMQ72		7
pKNT25 vector		Euromedex
pUT18 vector		Euromedex
pKT25-zip positive control		Euromedex
pUT18C-zip positive control		Euromedex
pKNT25 GcbC		6

pKNT25 GcbC (GGAAF)		6
pKNT25 GcbC R139E		This Study
pKNT25 GcbC R363E		8
pKNT25 GcbC R366E		8
pKNT25 GcbA		9
pKNT25 GcbB		9
pKNT25 WspR		9
pKNT25 Pfl01_0050		9
pKNT25 Pfl01_0190		9
pKNT25 Pfl01_0692		9
pKNT25 Pfl01_1323		9
pKNT25 Pfl01_1336		9
pKNT25 Pfl01_2049		9
pKNT25 Pfl01_2170		9
pKNT25 Pfl01_2176		9
pKNT25 Pfl01_2295		9
pKNT25 Pfl01_2297		9
pKNT25 Pfl01_3508		9
pKNT25 Pfl01_3550		9
pKNT25 Pfl01_3800		9
pKNT25 Pfl01_4084		9
pKNT25 Pfl01_4307		9
pKNT25 Pfl01_4451		9
pKNT25 Pfl01_5168		9
pUT18 GcbC		6
pUT18 LapD		6
pUT18 Pfl01_0192		9
pUT18 Pfl01_2920		9
**Primers**		
pMQ72 Sequence Verify Forward	5’ – CGCAACTCTCTACTGTTTCTCC – 3’	This Study
pMQ72 Sequence Verify Reverse	5’ – GGCTGAAAATCTTCTCTCATCCGCC – 3’	This Study
pKNT25 Sequence Verify Forward	5’ – GCGGATAACAATTTCACACAGG – 3’	This Study
pKNT25 Sequence Verify Reverse	5’ – CGTAACCAGCCTGATGCGATTGC – 3’	This Study
pKNT25-GcbC Forward	5’ – CGCACGCCAAGCTTGATGATCGCGAG – 3’	This Study
pMQ72-GcbC Forward	5’ – GGGCTAGCGAATTCGAGCTCGGTACCCCCGA AACCGAACCATGATCGCC - 3'	This Study
pMQ72-GcbC Reverse	5’ – GCAGGTCGACTCTAGAGGATCCCCTCAAGCGT	This Study

	AATCTGG – 3’	
GcbC R139A Point Mutation Forward	5’ – GCCGACGCTGACTACTTCCTCG – 3’	This Study
GcbC R139A Point Mutation Reverse	5’ – GTAGTCAGCGTCGGCGAAATTGC – 3’	This Study
GcbC R139E Point Mutation Forward	5’ – GCCGACGAAGACTACTTCCTCG – 3’	This Study
GcbC R139E Point Mutation Reverse	5’ – GTAGTCTTCGTCGGCGAAATTGC – 3’	This Study
GcbC R162A Point Mutation Forward	5’ – CCCGCTTGCGACTGCGATGACG – 3’	This Study
GcbC R162A Point Mutation Reverse	5’ – GCAGTCGCAAGCGGGCTTGAACG – 3’	This Study

Literature Cited

1. Simon R, Preifer U, Puhler A (1983) A broad host range mobilization system for *in vivo* genetic engineering: transposon mutagenesis in gram negative bacteria. *Nat Biotechnol* 1: 784-791.
2. Qi Y, Chuah MLC, Dong X, Xie K, Luo Z, Tang K, Liang Z (2011) Binding of cyclic diguanylate in the non-catalytic EAL domain of FimX induces a long-range conformational change. *J Biol Chem* 286: 2910-2917.
3. Friedman L, Kolter R (2004) Genes involved in matrix formation in *Pseudomonas aeruginosa* PA14 biofilms. *Mol Microbiol* 51(3): 675-690.
4. Monds RD, Newell PD, Gross RH, O'Toole GA (2007) Phosphate-dependent modulation of c-di-GMP levels regulates *Pseudomonas fluorescens* Pf0-1 biofilm formation by controlling secretion of the adhesin LapA. *Mol Microbiol* 63(3): 659-679.
5. Newell PD, Yoshioka S, Hvorecny KL, Monds RD, O'Toole GA (2011) Systematic analysis of diguanylate cyclases that promote biofilm formation by *Pseudomonas fluorescens* Pf0-1. *J Bacteriol* 193(18): 4685-4698.
6. Dahlstrom KM, Giglio KM, Collins AJ, Sondermann H, O'Toole GA (2015) Contribution of physical interactions to signaling specificity between a diguanylate cyclase and its effector. *mBio* 6(6):e01978-15. doi:10.1128/mBio.01978-15.
7. Shanks RMA, Calazza NC, Hinsa SM, Toutain CM, O'Toole GA (2006) *Saccharomyces cerevisiae*-based molecular tool kit for manipulation of gram-negative bacterial genes. *Appl Environ Microbiol* 72(7): 5027-5036.

8. Dahlstrom KM, Gilgio KM, Sondermann H, O'Toole HA (2016) The inhibitory site of a diguanylate cyclase is a necessary element for interaction and signaling with an effector protein. *J Bacteriol* 198(11): 1595-1603.

9. Dahlstrom KM, Collins AJ, Doing G, Taroni JN, Gauvin TJ, Greene CS, Hogan DA, O'Toole GA (2018) A multimodal strategy used by a large c-di-GMP network. *J Bacteriol*. doi:10.1128/JB.00703-00717.
